# Fitter Women Did Not Have Attenuated Hemodynamic Responses to Psychological Stress Compared with Age-Matched Women with Lower Levels of Fitness

**DOI:** 10.1371/journal.pone.0169746

**Published:** 2017-01-12

**Authors:** Sisitha U. Jayasinghe, Susan J. Torres, Mais Hussein, Steve F. Fraser, Gavin W. Lambert, Anne I. Turner

**Affiliations:** 1 School of Exercise and Nutrition Science, Institute for Physical Activity and Nutrition Research, Geelong, VIC, Australia; 2 Baker IDI Heart & Diabetes Institute, Melbourne, VIC, Australia; University of Debrecen, HUNGARY

## Abstract

According to the ‘cross stressor adaptation hypothesis’, regular exercise acts as a buffer against the detrimental effects of stress. Nevertheless, evidence that higher levels of cardiorespiratory fitness moderate hemodynamic responses to acute psychological stress is inconclusive, especially in women. Women aged 30–50 years (in the mid-follicular phase of the menstrual cycle) with higher (n = 17) and lower (n = 17) levels of fitness were subjected to a Trier Social Stress Test (TSST). Continuous, non-invasive measurements were made of beat-to-beat, systolic blood pressure (SBP), diastolic blood pressure (DBP), mean arterial pressure (MAP), heart rate (HR), stroke volume (SV), cardiac output (CO), left ventricular ejection time (LVET), maximum slope, pulse interval (PI) and total peripheral resistance (TPR). Maximal oxygen consumption was significantly (p<0.001) higher in the ‘higher fit’ women. Lower fit women had higher fasting glucose, resting heart rate, waist to hip ratios and elevated serum triglyceride and cholesterol/ HDL ratios compared with higher fit women (p<0.05 for all). While all measured parameters (for both groups)displayed significant (p<0.001) responses to the TSST, only HR, PI and LVET differed significantly between higher and lower fit women (p<0.001 for all) with the higher fit women having the larger response in each case. It was also found that higher fit women had significantly shorter time to recovery for maximum slope compared with the lower fit women. These findings provide little support for the notion that higher levels of cardiorespiratory fitness result in lower cardiovascular responsivity to psychological stress in women but may indicate that lower fit women have blunted responses to stress.

## Introduction

Exposure to psychological stress can rapidly activate the autonomic nervous system wherethe activation of the sympathetic nervous system (SNS) can result in numerous cardiovascular responsesincluding a rise in heart rate, blood pressure, cardiac output and total peripheral resistance [1]. Physiological responses to psychological stressors are generated due to anticipation of a possible disruption of physiological homeostasis, rather than to a genuine threat. Therefore, the stress response generated in such instances is “anticipatory” [2]. While it is essential to sufficiently activate the SNS in response to stress (to facilitate an adaptive response to the stressor encountered), unnecessary over activation of the SNS can be detrimental and may lead to chronic diseases including cardiovascular disease, type 2 diabetes and psychological disorders [3–8].

The cross stressor adaptation hypothesis states that ‘A stressor of sufficient intensity and/or duration will induce an adaptation of the stress response systems, which becomes apparent in other similarly taxing states’[9]. In agreement with this hypothesis,higher levels of cardiorespiratory fitness achieved via physical exercise, acts as a buffer against the detrimental effects of stress [10–12]. Exercise, upon repetition is thought to elicit counterconditioning to other stimuli perhaps through mechanisms such as the central catecholamine system and the central/peripheral opioid system [13]. The general consensus is that regular exercise that increases cardiorespiratory fitness is associated with an attenuation of cardiovascular responses to a wide variety of stressors[9, 10, 14, 15]. Nevertheless, the evidence regarding cardiorespiratory fitness and cardiovascular system reactivity in response to psychological stress remains inconclusive, especially in women. For instance, there are studies that have found both beneficial effects of fitness (i.e. reduction in heart rate and blood pressure responses to stress) [16–21], as well as no influence of fitness on cardiovascular responses to stress[22–24]. In a recent study conducted by our research group, we showed that increased cardiorespiratory fitness did not moderate cortisol, catecholamine or cardiovascular responses to psychological stress (Trier Social Stress Test; TSST) in 30–50 year old women who had varying levels of physical fitness[25]. However, cardiovascular parameters were only measured every 7–15 minutes so it is possible that some outcomes were not detected. It is also important to acknowledge that there is an increasing body of evidence that suggests that blunted cardiovascular and endocrine responses to acute stress may also be associated with ill health [26]. By recruiting seemingly healthy women we expect to have minimised instances of a ‘blunted’ stress response from this study. Nevertheless, reduced responses to stress may be indicative of poorer health outcomes.

The inconclusive results in previous experiments may be due to variations in methodologies that have been implemented. A wide variety of stressors such as mental arithmetic with auditory distraction, competitive motor tasks and competitive reaction time tasks [18, 27, 28]were used in previous experiments whichmay not have been capable of eliciting a robust and repeatable autonomic nervous system response as occurs when using the TSST[29–32]. In addition, previous studies lack continuous measuremen tof cardiovascular parameters. The rapidly changing nature of the cardiovascular response necessitates frequent sampling to capture the finer details ofthe response patterns. This may also provide a more holistic view of the cardiovascular responses to stress. To the best of our knowledge, a complete hemodynamic profile has not been measured in experiments that have investigated the effects of physical activity levels on cardiovascular responses to psychological stress. In addition, fewer studies have investigated these responses in women[14]. Given the known sex differences in stress responsiveness [33], findings in males are not necessarily generalisableto females.

The aim of this study was to investigate the impact of cardiorespiratory fitness onhemodynamic responses to a TSST using continuous non-invasive sampling in 30–50 year old women. A complete profile of hemodynamic responses was assessed through the measurement of systolic blood pressure (SBP), diastolic blood pressure (DBP), mean arterial pressure (MAP), heart rate (HR), stroke volume (SV), cardiac output (CO), left ventricular ejection time (LVET), maximum slope, pulse interval (PI) and total peripheral resistance (TPR). It was hypothesised that women who have higher levels of cardiorespiratory fitness will have lower hemodynamic cardiovascular responsivity compared with age matched women who have relatively lower levels of cardiorespiratory fitness.

## Materials and Methods

Healthy women with no history or current metabolic disease (n = 34;aged 30–50 years) were recruited from the Melbourne metropolitan area, Victoria, Australia using several methods including news paper and online advertisements, emails, fliers in community centres and medical clinics. Women were excluded if they had current or prior diagnosis/incidence of: Cushing’s syndrome, any stress or anxiety disorder, depression, any diseases of the adrenal gland, type 2 diabetes, heart disease, elevated total cholesterol (>5.5 mmol/L), stroke, cancer (including cancer remission) or any other disorder that may potentially impact the stress pathway activity. None of the women were medicated at the time of recruitment or at the time of either testing session. Given the influence of sex steroids on stress pathway activity, women were also excluded if they were post-menopausal, peri-menopausal or were on any form of steroidal contraception (including oral contraceptives, steroidal implants and steroidal intrauterine devices) [33].

All procedures implemented in this research were approved by the Human Research Ethics Committee of Deakin University (Project code: 2011–242) and conformed to the guidelines of the National Health and Medical Research Council’s National Statement on Ethical Conduct in Human Research (2007). Participants were also required to provide informed consent prior to commencing the study.

### Experimental procedure

All testing was conducted at our Clinical Research Facility in the School of Exercise and Nutrition Sciences at Deakin University. Each participant attended our facility on two separate testing days. Details of the procedures undertaken on each testing day are included below. The second testing day occurred at least one week after the first.

### Day 1 testing

A fasting blood sample for the measurement of cardio-metabolic risk markers was collected and maximum oxygen consumption (VO_2_max) was determined. Participants were given instructions to fast overnight (for at least 10 hours) prior to attending the laboratory. Body weight (TANITA, Wedderburn, Melbourne, Australia), height (Measurement Concepts, North Bend, Australia), BMI (weight/ (height in meters)^2^) and resting blood pressure (Criticare systems Inc, Wisconsin, USA) were recorded. Women whose BMI fell outside the range 18–30 (kg/m^2^) or whose resting blood pressure exceeded 140mmHg for SBP or 90 mmHg for DBP were excluded. A blood sample (GreinerBio-One GmbH, Kremsmunster, Austria) was collected from all eligible women through standard venepuncture procedures for the analysis of cardiometabolic risk markers (total cholesterol, high density lipoprotein, low density lipoprotein, triglycerides, fasting serum glucose, C-reactive protein and fasting insulin).

The women were then offered an optional snack (a selection of foods from muesli bars, nuts, dried fruit and juice boxes were made available) and further gathering of information was undertaken via a Physical Activity Readiness Questionnaire (PAR-Q), an International Physical Activity Questionnaire (IPAQ) [34], a State-Trait Anxiety Inventory (STAI) [35] and a Beck Depression Inventory (BDI-ii) [36]. Information from the PAR-Q (confirming suitability to undertake vigorous intensity exercise) was used as an eligibility criterion priorto undertakinga VO_2_max test. A thorough explanation of the requirements of the fitness test was provided to all participants after the snack. This was followed by a graded VO_2_maxtest according to the methods that have been previously used by our research group [25]. After ranking women by VO_2_max score, a median split was used to allocate women evenly into a group with high VO_2_max (higher fit group; n = 17) and a group with low VO_2_max (lower fit group; n = 17) [37]. Water was available *ad libitum* to all participants throughout the testing session.

### Day 2 testing

To standardise the influence of stage of menstrual cycle on results, this session was conducted in the mid follicular phase (Days 5–9) of the participants’ menstrual cycle[38]. This time frame for the mid follicular phase is consistent with multiple previous reports that investigated the relationship between phase of the menstrual cycle and the activity of the stress pathways [39–42]. All womenwere asked to abstain from smoking, ingesting any caffeine containing beverages (e.g. tea, coffee, cola), liquorice, alcohol or drugs (except for any regular medications) and from strenuous physical activity during the 12 hours prior to participation in the Stress testing session.

**Fig 1:**
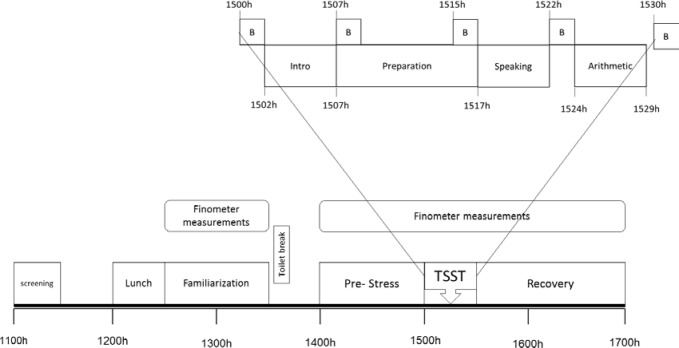
Schematic representation of the stress testing day. TSST = Trier Social Stress Test, B = biological samples (results from biological samples are reported elsewhere [25]).

The experimental procedure for Day 2 is shown in Fig 1. Participants arrived at the laboratory at 1100h. Anthropometric measures (waist and hip circumference andbio impedance estimation of body fat [TANITA, Wedderburn, Melbourne, Australia]) were obtained between 1100h-1145h. Waist circumference was measured at the midpoint between the last rib and the anterior superior iliac spine, using a tape measure, and hip circumferencewas measured at the widest point of the gluteal area[43]. A questionnaire regarding smoking status, alcohol consumption and physical activity in the week leading up to the testing sessionwas completed by the participants during this time. Astandard lunch containing approximately 20% protein, 61% carbohydrate and 19% fat was consumed by all participants between 1200h-1230h. As previously reported by our group there were no differences in the cortisol, blood pressure and heart rate responses in higher and lower fit women in response to the standardised lunch [44]. This was followed by the sampling procedures which began at 1230h. Continuous beat-to-beat SBP, DBP, HR,CO, TPR, LVET, SV, PI and maximum slope were measured using aFinometer (Finometer model-1 (PRO); Finopres Medical Systems BV, Amsterdam, the Netherlands) attached to a finger on the non-dominant arm. This unit also contained sensors for the adjustment of movement artefacts.A period of familiarisation with the sampling procedures took place between 1230h-1330h. Participants were given a break at 1330h in which they made avisit to the restroom. Further beat-to-beat monitoring was undertaken from 1400h to 1700h. A TSST was imposed between 1500h-1530h (details below) and a recovery period was monitoredfrom 1530h-1700h.

### TSST

Psychological stress was induced using a modified version [45] of the well characterised Trier Social Stress Test (TSST) protocol[29]. The modifications included a 2 personnel (instead of 3) interviewing panel, a 60-minute lead in time prior to exposure to stress and biological sampling in-between the public speaking task and the mental arithmetic challenge. At 1500h, participants were introduced to a panel and were given instructions about a speaking task to follow (Fig 1, inset). This was followed by a 10min preparation phase and then five minutes of public speaking. Further instructions followed regarding a mental arithmetic task and then 5 min of mental arithmetic was undertaken. Continuous cardiovascular parameter measurements using the Finometer were made throughout this entire period.

### Statistical analysis

#### Preliminary analysis

Beat-to-beat monitoring of cardiovascular information from 1400h-1700h resulted in approximately 15000–16000 data points for each subject. For clarity of data presentation, means of 2 minute blocks were calculated for the entire sampling period. Data from 1400–1430h were excluded from the final analysis due to complications (such as missing data) during the initial 30 minutes of sampling. This resulted in 15, 16 and 44 time points for pre-treatment, stress and recovery phases, respectively.

**Pre-treatment** for all measured parameters was defined as the average of means of the 15, 2-minute blocks from 1430h-1500h. **Peak height** for blood pressures, HR and CO was defined as the highest value obtained between 1500h-1600h inclusive. Since some cardiovascular parameters (LVET, SV and pulse interval) decrease rather than increase in response to acute stress, **lowest trough** for LVET, SV and pulse interval was defined as the lowest reading recorded between 1500h-1600h inclusive. This variable is the equivalent of peak height but is used for parameters that decrease instead of increasing in response to acute stress. **Reactivity** was calculated as the difference between pre-treatment and peak height/ lowest trough for all parameters. **Area under the curve** (with respect to increase) was calculated for each parameter using values from 1500 to 1700 h after the subtraction of the pre-treatment value from each data point[46]. Areas under the curve were calculated using the trapezoid rule utilising Sigmaplot graphing software (SystatSoftwareInc., California, USA).

As described by our group previously [45], **recovery time** for all parameters was defined as the time difference from the commencement of the stressor (1500h) to the point at which the relevant parameter returned to within two standard deviations of its pre-treatment value. Those who did not have a significant response to the stressor (i.e. those who did not exceed two standard deviations of the pre-treatment value between 1500–1700h; n = 1 [PI] lower fit, n = 1, 1 [LVET] lower/higher fit, n = 1, 1 [SV] lower/higher fit, n = 5, 1 [CO] lower/higher fit, n = 3 [TPR] lower fit, n = 1 [max slope] lower fit) were excluded from analyses of recovery time. For those who did exceed two standard deviations but did not return to within two standard deviations by 1700h (n = 1, 1 [SBP] lower/higher fit, n = 2, 2 [DBP] lower/higher fit, n = 1, 2 [MAP] lower/higher fit, n = 1 [SV] lower fit, n = 2, 3 [TPR] lower/higher fit, n = 1 [max slope] lower fit), 120min was used as the recovery time in the analyses.

#### Analysis

Data were analysed using the Statistical Package for the Social Sciences software version 20.0 (SPSS. Inc, Chicago, USA). Kolmogorov-Smirnov and Shapiro–Wilk tests were conducted to test for normality. Tests for homogeneity of variance were conducted using Levene’s test of equality of error variances. Descriptive characteristics were compared between groups using univariate analysis of variance. Cardiovascular parameters were compared within and between groups using repeated measures analysis of variance. The within subjects factor was time and the between subjects factor was cardiorespiratory fitness (higher fit vs lower fit). Derived cardiovascular parameters (pre- treatment, peak height/lowest trough, reactivity, area under the curve and recovery time) were compared between groups using univariate analysis of variance. Pearson’s correlation without adjustment and with adjustments (partial correlations) for central adiposity (by means of WHR) and metabolic parameters (by means of HOMA-IR) were conducted between VO_2_ max and reactivity and area under the curve of all dependant variables.

## Results

### Participants

Data from 34 women are presented in this study. The data presented in this paper are part of a larger study (17) which assessed cortisol and catecholamine responses to psychological stress in women. A total of 44 women were tested in the larger study. Nevertheless, ten women had to be excluded from the current analysis due to incomplete data.The remaining 34 women,used in this study,were ranked by VO_2_ max and a median split was used to allocate women to either the lower fit (n = 17) or the higher fit group (n = 17). The 10 women who were excluded from the larger study did not differ from the final cohort in any of the descriptive characteristics (data not shown). Trait anxiety score, insulin data and HOMA-IR were unavailable for two women (both from the higher fit group).

### Participant characteristics

VO_2_ max was significantly higher in women in the higher fit group compared with the women in the lower fit group (Table 1). As expected, VO_2_ max and the number of hours of physical activity per week were significantly positively correlated (r = 0.428; p = 0.012). Both groups were in the healthy weight range and had similar BMI levels, body fat percentages, waist circumference and hip circumference (Table 1). However, WHR was significantly higher (p = 0.041) in the lower fit women compared with the higher fit women (Table 1). Resting heart rate (p = 0.048) and diastolic blood pressure (p = 0.004) were also higher in the lower fit women compared with the higher fit women (Table 1). Resting SBP, resting SBP variability and resting DBP variability were comparable between low and high fit women (Table 1). Triglycerides (p = 0.034), fasting glucose (p = 0.045) and CHOL/HDL ratio (p = 0.005) were significantly higher in the lower fit women compared with the higher fit women (Table 2). All other cardio metabolic risk factors and depression/anxiety scores were comparable between the groups (Table 2).

**Table 1:** Mean (±SEM) descriptive characteristics of Lower and Higher fit women.

	Lower fit (n = 17)	Higher fit (n = 17)	P value*
Age (years) at screening	39.5±1.7	37.3±1.4	0.319
Hours of physical activity per week	4.1±1.1	5.1±0.6	0.414
VO_2_max (mL/kg/min)	27.6±1.2	41.5±1.7	**<0.001**
Weight (kg)	62.0±2.7	62.7±1.9	0.832
BMI (kg/m^2^)	23.0±0.8	22.3±0.5	0.450
Body fat (%)	29.4±1.6	25.8±1.4	0.108
Waist circumference (cm)	81.2±2.7	76.5±1.6	0.138
Hip circumference (cm)	97.0±1.9	96.4±1.3	0.801
Waist to hip ratio	0.84±0.0	0.79±0.0	**0.041**
Resting HR (bpm)	71±2	65±3	**0.048**
Resting SBP (mmHg)	116±3	113±3	0.605
Resting SBP variability (SD)	6.0±1	6.9±2	0.632
Resting DBP (mmHg)	74±2	65±2	**0.004**
Resting DBP variability (SD)	4.4±1	6.9±2	0.132

* Univariate Analysis of Variance, HR = heart rate, SBP = Systolic Blood Pressure, DBP = Diastolic Blood Pressure.

**Table 2:** Mean (±SEM) cardio-metabolic risk markers and depression and anxiety scores of Lower and Higher fit women.

	Lower fit	Higher fit	P value*
CRP (mg/L)	1.6±0.9	0.5±0.2	0.242
Cholesterol (mmol/L)	5.1±0.2	4.8±0.2	0.293
Triglycerides (mmol/L)	1.0±0.1	0.8±0.1	**0.034**
CHOL/HDL ratio	3.2±0.2	2.6±0.1	**0.005**
Fasting glucose (mmol/L)	5.3±0.1	5.0±0.1	**0.045**
Insulin (μU/mL)	12.8±1.2	12.0±0.5	0.512
HOMA-IR	3.0±0.3	2.7±0.1	0.328
BDI-ii score	3.9±1.0	3.1±1.2	0.606
STAI score (trait)	31.8±1.7	32.4±2.4	0.819
STAI score (state)	32.3±1.4	30.8±2.6	0.621

* Univariate Analysis of Variance, BDI = Beck Depression Inventory, STAI = State-Trait Anxiety Inventory, HOMA-IR = Homeostatic Model Assessment- Insulin Resistance.

n = 15 for STAI score (trait), insulin and HOMA-IR in the Higher fit group. n = 17 in all other instances.

### HR, PI and LVET

**Fig 2:**
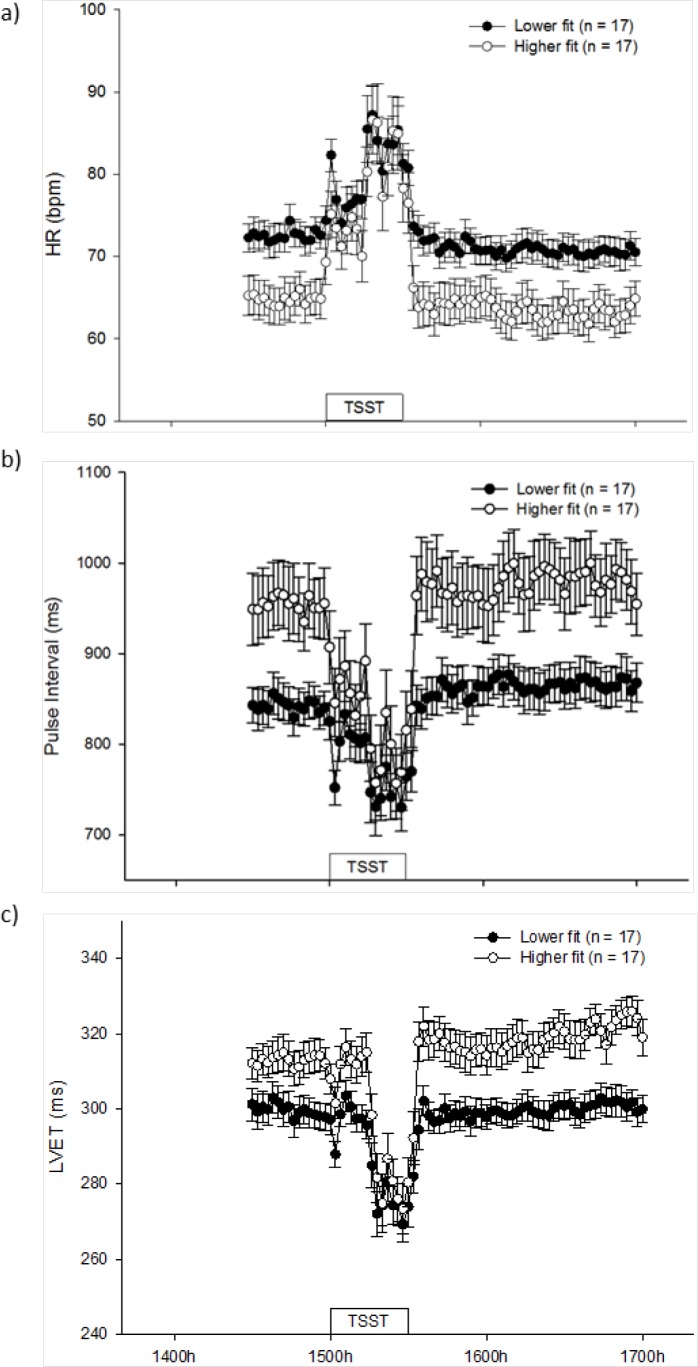
Mean (± SEM) heart rate (2a), pulse interval (2b) and left ventricular ejection time (2c) in lower and higher fit women from 1430h-1700h. TSST: Trier Social Stress Test.

HR, PIand LVET in lower fit and higher fit women are shown in Fig 2A, 2B and 2C, respectively and Table 3. Repeated measures analysis of variance revealed that there was a significant effect of time for all three parameters (p<0.001 for all; Fig 2A, 2B and 2C). Overall (both groups combined), the peak height of HR (93±3 bpm) and lowest troughs of PI (680±20 ms) and LVET (262±4 ms) were significantly higher/lower than their respective pre-treatment values (69±2 bpm, 899±22 ms and 306±3 ms) (p<0.001). Overall, there was a 35% increase in HR, an11% decrease in PI and a14% decrease in LVET from pre-treatment values to the highest peak/lowest trough of the response (both groups combined).

**Table 3:** Mean (±SEM) pre-treatment, peak height/lowest trough, reactivity, area under the curve and recovery time for HR, LVET and Pulse interval in lower and higher fit women.

	Lower fit (n = 17)	Higher fit(n = 17)	p value*
HR			
Pre-treatment (bpm)	73±2	65±2	**0.008**
Peak height (bpm)	93±3	93±4	0.937
Reactivity (bpm)	20±2	28±3	**0.031**
AUC (bpm *min)	91±72	374±75	**0.010**
Recovery time (min)	30±3	31±2	0.825
Pulse interval			
Pre-treatment (ms)	842±20	955±35	**0.009**
Lowest trough (ms)	672±20	688±34	0.677
Reactivity (ms)	-171±18	-266±23	**0.003**
AUC (ms *min)	-301±878	-2914±937	**0.050**
Recovery time (min)	29±3^#^	28±2	0.835
LVET			
Pre-treatment (ms)	300±4	313±4	**0.025**
Lowest trough (ms)	259±5	266±6	0.398
Reactivity (ms)	-41±6	-47±5	0.368
AUC (ms *min)	-262±262	-584±310	0.433
Recovery time (min)	38±5^#^	33±1^#^	0.327

* Univariate Analysis of Variance

^#^n = 16

AUC = Area under the curve.

In response to the TSST, HR, PI and LVET all differed between lower fit women and higher fit women (time*treatment, p<0.001 for all; Fig 2A, 2B and 2C).In keeping with this finding, reactivity and AUC for HR and PI were significantly larger in higher fit women than in lower fit women (p≤0.05 for all; Table 3). Nevertheless, reactivity and AUC for LVET did not differ significantly between the groups (Table 3).There were no significant differences between groups in peak height/lowest trough or time to recovery in any of the three parameters (Table 3). Significant between subjects effects in HR, PI and LVET were evident for all three variables (p< 0.05 for all)indicating that there were significant overall differences between the groups. This was reflected in pre-treatment values for HR being significantly lower in higher fit women and in pre-treatment values for PI and LVET being significantly higher in higher fit women (p<0.05 for all; Fig 2).

### SBP, DBP and MAP

**Fig 3:**
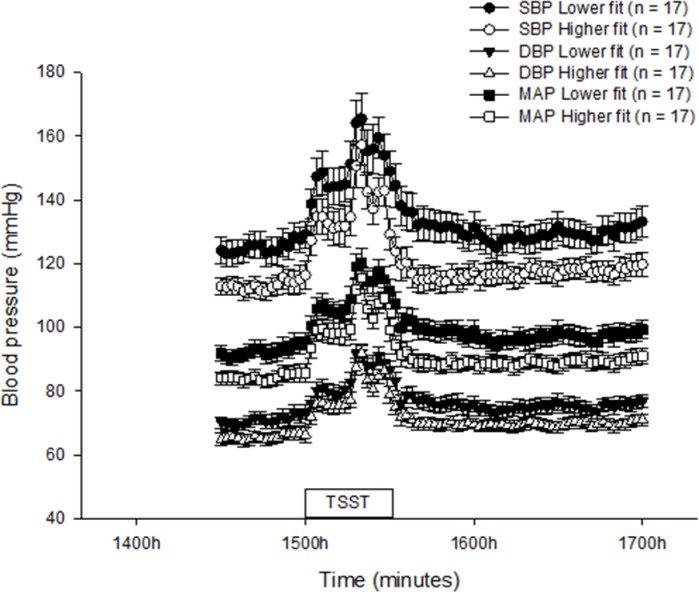
Mean (± SEM) SBP, DBP and MAP in lower and higher fit women from 1430h-1700h. TSST: Trier Social Stress Test.

All blood pressure parameters in lower fit and higher fit women are shown in Fig 3. Repeated measures analysis of variance revealed that there was a significant effect of time for all blood pressure parameters (p<0.001 for all; Fig 3). Overall (both groups combined), the peak heights of SBP (165±5 mmHg), DBP (94±2 mmHg) and MAP (121±3 mmHg) were significantly higher than their respective pre-treatment values (119±3, 68±1 and 88±2 mmHg;p<0.001 for all). Overall, there was a 39% increase in SBP, a 38% increase in DBP and a 36% increase in MAP from pre-treatment pressures to the peak of the response (both groups combined).

None of the blood pressure parameters in response to the TSST differed between lower fit women and higher fit women (time*treatment, p = 0.854 (SBP), p = 0.997 (DBP) and p = 0.996 (MAP); Fig 3) and accordingly, there were no significant differences between the groups in peak height, reactivity, area under the curve or mean time to recovery for the blood pressure responses (data not shown). A significant between subjects effect indicated that overall, SBP, DBP and MAP were significantly higher (p = 0.025, 0.040 and 0.020 for SBP, DBP and MAP; respectively) in the lower fit women compared with higher fit women(Fig 3). This was reflected in pre-treatment values for all parameters which were significantly higher (p = 0.019 [SBP], p = 0.054 [DBP] and p = 0.014 [MAP]) in the lower fit women compared with thehigher fit women (125±4 vs 113±3 [SBP], 71±2 vs 66±2 [DBP] and 92±3 vs 84±2 [MAP] mmHg, respectively).

### SV, CO, TPR and maximum slope

**Fig 4:**
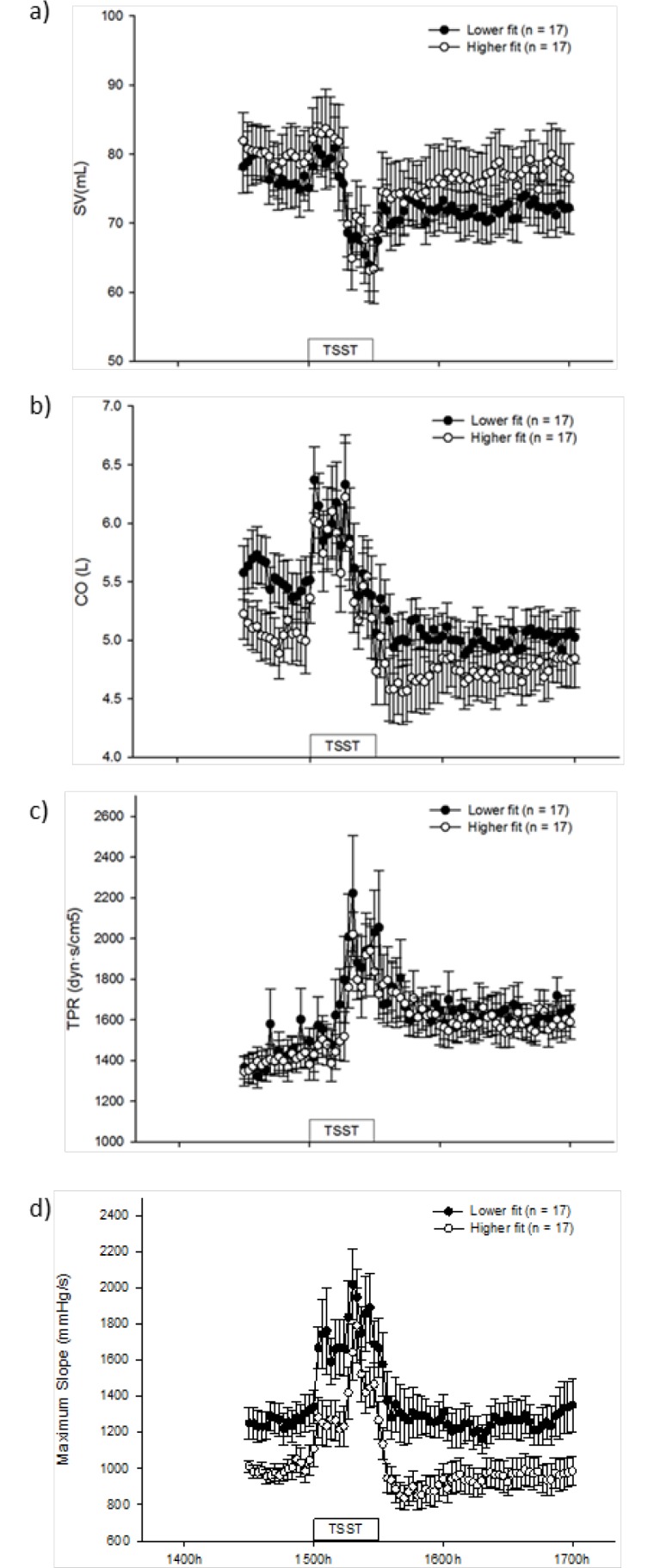
Mean (± SEM) stroke volume (4a), cardiac output (4b), total peripheral resistance (4c) and maximum slope in lower and higher fit women from 1430h-1700h. TSST: Trier Social Stress Test.

SV, CO, TPR and maximum slope in lower fit and higher fit women are shown in Fig 4A, 4B, 4C and 4D, respectively. Repeated measures analysis of variance revealed that there was a significant effect of time for all of the aforementioned parameters (p<0.001 for all; Fig 4). Overall (both groups combined), the lowest trough of SV (55±2 mL) and the peak height of CO (7±0.3L), TPR (2499±173dyn·s/cm^5^) and max slope (2079±155 mmHg/s) were significantly lower/higher than their respective pre-treatment values (79±3 mL, 5±0.2 L, 1407±45 dyn·s/cm^5^and 1129±52 mmHg/s) (p<0.001). Overall, there was a 29% decrease in SV, a 32% increase in CO, a 77% increase in TPR and an84% increase in max slope from pre-treatment values to the lowest trough / highest peak of the response (both groups combined).

None of these parameters differed between the lower fit women and higher fit women in response to the TSST(time*treatment, p = 0.956 (SV), p = 0.953 (CO), p = 0.791 (TPR) and p = 0.609 (max slope); Fig 4) and accordingly, there were no significant differences between the groups in peak height/lowest trough, reactivity or area under the curve for any of these parameters (data not shown). The mean time to recovery was comparable between the groups for SV, CO and TPR, but mean time to recovery for max slope was significantly longer(p = 0.048) in the lower fit women compared with the higher fit women (41±6 and 28±2 min, respectively). There was no significant between subjects effect in SV, CO or TPR indicating that there were no significant overall differences between the groups for any of these variables (p = 0.512, 0.332 and 0.500 for SV, CO and TPR; respectively). Nevertheless, there was a significant overall difference (p = 0.016) between the groups in max slope (Fig 4D) with lower fit women having a significantly higher max slope overall than higher fit women. This was reflected in there being a significantly higher (p = 0.008) max slope during pre-treatment in the lower fit women compared with the higher fit women (1262±82 and 995±49 mmHg/s, respectively).

### Correlations/ Associations

In unadjusted correlations, there were significant (P<0.05) associations between VO_2_max and the reactivity and AUC for heart rate and pulse interval but no associations with any of the other cardiovascular parameters (Table 4). When adjustment was made for central adiposity (by means of WHR) and metabolic parameters (by means of HOMA-IR), a significant association was seen between VO_2_max and the reactivity and AUC for mean arterial pressure and between VO_2_max and the AUC for cardiac output (Table 4),

**Table 4:** Associations between VO_2_max and reactivity and area under the curve (AUC) for cardiovascular parameters.

	Unadjusted correlations with VO_2_max		Adjusted ^a^ correlations with VO_2_max	
	Pearson’s r	p-value	Pearson’s r	p-value
HR reactivity	**0.391**	**0.025**	0.288	0.116
HR AUC	**0.362**	**0.038**	**0.379**	**0.035**
PI reactivity	**-0.594**	**<0.001**	**-0.514**	**0.003**
PI AUC	**-0.419**	**0.015**	**-0.436**	**0.014**
LVET reactivity	-0.120	0.507	-0.020	0.915
LVET AUC	0.077	0.672	0.075	0.688
SBP reactivity	-0.103	0.568	0.172	0.354
SBP AUC	-0.152	0.399	-0.052	0.781
DBP reactivity	0.075	0.680	0.327	0.072
DBP AUC	0.065	0.719	0.301	0.100
MAP reactivity	0.002	0.990	**0.379**	**0.035**
MAP AUC	0.081	0.652	**0.374**	**0.038**
SV reactivity	-0.039	0.828	-0.076	0.685
SV AUC	0.133	0.461	0.164	0.378
CO reactivity	0.319	0.070	0.337	0.064
CO AUC	0.319	0.070	**0.360**	**0.047**
TPR reactivity	-0.187	0.298	-0.140	0.451
TPR AUC	-0.152	0.399	-0.052	0.781
Max slope reactivity	0.013	0.944	0.025	0.893
Max slope AUC	0.065	0.721	0.082	0.660

HR = heart rate, PI = pulse interval, LVET = left ventricular ejection time, SBP = systolic blood pressure, DBP = diastolic blood pressure, MAP = mean arterial pressure, SV = stroke volume, CO = cardiac output, TPR = total peripheral resistance, Max slope = maximum slope; statistically significant results shown in bold; n = 33

^a^ adjustment was made for levels of central adiposity (WHR) and metabolic parameters (HOMA-IR).

## Discussion

This study found that women who had higher levels of cardiorespiratory fitness (higher fit women) had greater HR, LVET and PI responses to a potent psychological stress compared with aged-matched women who had lower levels of cardiorespiratory fitness (lower fit women).It was also found that higher fit women had a significantly shorter time to recovery for maximum slope compared with the lower fit women. Furthermore, after adjustment for central adiposity and metabolic parameters, the response of mean arterial pressure (reactivity and AUC) and cardiac output (AUC) was found to be positively associated with VO_2max_ indicating that higher fit women had higher responses of these parameters when controlling for covariates. All other parameters (SBP, DBP, SV and TPR) were comparable between the groups in response to the TSST. This suggests that cardiovascular and hemodynamic responses to psychological stress are only partially influenced by the level of cardiorespiratory fitness. While the current results mostly suggest an association between higher levels of cardiorespiratory fitness and heightened activity of the cardiovascular system (indicated by greater HR, LVET, PI, MAP and CO) in response to psychological stress, the rapid recovery of maximum slope in higher fit women suggests there may be some improvement of response in the higher fit women albeit a very subtle effect. These findings provide little support for the notion that women with higher levels of cardiorespiratory fitness will have lower cardiovascular responsivity to psychological stress compared with women who have relatively lower levels of cardiorespiratory fitness. Nevertheless, these findings may indicate that lower fit women have reduced or blunted HR, LVET, PI, MAP and CO responses to stress compared with high fit women.

Both higher and lower fit women reached similar HR peaks in response to the TSST. These peaks are well within the range of HR responses to TSSTs reported before [47]. However, it is evident in the current study that the higher fit women had a lower HR before the commencement of the stress testdespite peaking at the same level as the lower fit women during exposure to psychological stress. As such, there may be a “ceiling” effect with HR which prevents it from further elevations in either group. This provides the appearance of an accentuated HR response in higher fit women. Nevertheless, this could merely be a result of the higher fit women having a lower baseline thus overall, still having the healthier response.A similar response was observed in one of our previous studies in lean and overweight/obese men [1]. In that study, lean men had a lower HR at the commencement of the TSST, but reached a maximum that was comparable to the overweight/obese men after exposure to stress.However, the findings in the present study where the HR reactivity was greater in the higher fit women compared to lower fit women disagree with other published findings. Traustadottir et al. 2005 reported greater HR reactivity to a Matt Stress Reactivity Protocol in young unfit individuals compared with older fit and unfit women. Higher HR responses were also reported in inactive young women in comparison to their more active counterparts [48] and more recent findings in men show lower HR responses to a TSST after improving fitness through exercise intervention [21]. While it is not consistent with the results of the studies listed above, an alternative way of considering our data may be that the fitter women are having a normal response to the stressor while lower fit women are having a reduced or blunted response. As indicated earlier, reduced responsivity of the stress systems may also be associated with ill health [26].

Our finding that the response of mean arterial pressure (reactivity and AUC) and cardiac output (AUC) were significantly positively associated with VO_2max_ after adjustment for central adiposity and metabolic parameters may indicate a similar circumstance to that of heart rate. With fitter women starting from a lower baseline for these variables, it is possible that they have reached a similar “ceiling” of response as the lower fit women. Nevertheless, these findings are not consistent with those of Palatini and colleagues who showed a higher blood pressure response in sedentary hypertensive individuals in response to a public speaking task compared with both active hypertensive individuals and controls [49]. As with heart rate, an alternative may be that the lower fit women are displaying a blunted response for mean arterial pressure and cardiac output.

The successful execution of a stress experiment depends on the ability of the stressor utilised to elicit a substantial reaction from the physiological stress pathways that are being investigated. In the current study, all measured parameters showed significant changes in response to the TSST. The various components of the TSST have shown to generate substantial cardiovascular responses consistently in comparison to executing a solitary stressor [50]. However, it is possible that our modified version of the TSST used in the current experiment may have maximally acutely activated the cardiovascular system in both groups. As such, it is possible that, had a more subtle (sub-maximal) stressor been used, the lower fit women may indeed have shown a larger response compared with the higher fit women.

Alternatively, it is also possible that the pattern of results observed in the current study is an indication that cardiorespiratory fitness in females may bare less impact on reducing excessive cardiovascular responses to psychological stresscompared with men. Previous reports by Rimmele and colleagues have shown that in men, elevated levels of cardiorespiratory fitness are associated with attenuated cardiovascular responses (lower HR) to psychological stress[16, 17]. We did not observe an obvious attenuation of cardiovascular reactivity pattern in the higher-fit women in the current experiment. Therefore, it could be speculated that attenuation in cardiovascular reactivity in response to psychological stress mediated by fitness status is influenced by sex and sex steroids.

As mentioned in the introduction[26], there is evidence that supports the notion of a blunted physiological response to acute stress being associated with symptoms related to ill health including, but not limited to, obesity[51], depression[52] and poorer cognitive function [53]. Reasons for a blunted reaction to acute stimuli may include reduced physiological capacity to respond to a given stimulus and a variety of psychological reasons[26].All women who participated in the current experiment were seemingly healthy, and all of them were capable of eliciting a substantial response to the TSST. Furthermore, based on the depression and anxiety levels (judged by the BDI-11 and STAI), there is no grounds to speculate that the women included in this study were psychologically compromised. Nevertheless, it is possible that the lower level of response seen in the lower fit women was indicative of a reduced ability to mount a normal stress response. Hypo-responsivity of the stress response such as this may be associated with poorer health outcomes [26].

Aside from the responses to psychosocial stress in lower and higher fit women, it should be noted that the lower fit women had a poorer cardiovascular profile overall than higher fit women. Overall, lower fit women had higher HR, SBP, DBP, MAP and maximum slope and lower PI and LVET compared with higher fit women. When you consider that these women also had higher waste to hip ratio, triglycerides, CHOL/HDL ratio and fasting glucose than higher fit women, it is clear that the overall health profile of these women was reduced compared with that of the higher fit women.

A significant strength of the current experiment is the continuous measurement of beat to beat cardiovascular changes. This measurement strategy, which has been validated for the use in cardiovascular research [54, 55],enables precise detection of cardiovascular changes and provides a more holistic view of the stress response [56]. We also utilised a modified version of the TSST which elicited substantial cardiovascular reactions in both groups of women. Future research could include women with much larger differences in fitness levels and women who are in different phases of their menstrual cycles. It is also noteworthy that the fitness levels of the women who took part in this study were representative of the prevailing fitness norms of 30–50 year old women [57]. Nevertheless, the authors acknowledge that exercise dependence was neither measured nor controlled for in the current study. Such measurements were beyond the scope of this study.

According to the ‘cross stressor adaptation hypothesis’, regular physical exercise acts as a buffer against the detrimental effects of stress. We tested the hypothesis that women who have higher levels of cardiorespiratory fitness will have lower cardiovascular responsivity compared with age matched women who have relatively lower levels of cardiorespiratory fitness. Nevertheless, results reported and discussed in this paper provide little support for the notion that higher levels of cardiorespiratory fitness result in lower cardiovascular responsivity to psychological stress in women. Instead, lower fit women may have had a blunted response to stress. More investigations need to be conducted to elucidate the exact workings behind the relationship between cardiorespiratory fitness and cardiovascular responses to stress.

## Supporting Information

S1 Data Cumulative data.xlsx. (XLSX)
